# Structural insights into the biological functions of the long pentraxin PTX3

**DOI:** 10.3389/fimmu.2023.1274634

**Published:** 2023-10-09

**Authors:** Anna Margherita Massimino, Filippo Emanuele Colella, Barbara Bottazzi, Antonio Inforzato

**Affiliations:** ^1^ Department of Biomedical Sciences, Humanitas University, Pieve Emanuele, Italy; ^2^ Laboratory of Cellular and Humoral Innate Immunity, IRCCS Humanitas Research Hospital, Rozzano, Italy

**Keywords:** pattern recognition molecules (PRM), pentraxins, PTX3, structure, complement, extracellular matrix

## Abstract

Soluble pattern recognition molecules (PRMs) are a heterogenous group of proteins that recognize pathogen- and danger-associated molecular patterns (PAMPs and DAMPs, respectively), and cooperate with cell-borne receptors in the orchestration of innate and adaptive immune responses to pathogenic insults and tissue damage. Amongst soluble PRMs, pentraxins are a family of highly conserved proteins with distinctive structural features. Originally identified in the early 1990s as an early inflammatory gene, PTX3 is the prototype of long pentraxins. Unlike the short pentraxin C reactive protein (CRP), whose expression is mostly confined to the liver, PTX3 is made by several immune and non-immune cells at sites of infection and inflammation, where it intercepts fundamental aspects of infection immunity, inflammation, and tissue remodeling. Of note, PTX3 cross talks to components of the complement system to control cancer-related inflammation and disposal of pathogens. Also, it is an essential component of inflammatory extracellular matrices (ECMs) through crosslinking of hyaluronic acid and turn-over of provisional fibrin networks that assemble at sites of tissue injury. This functional diversity is mediated by unique structural characteristics whose fine details have been unveiled only recently. Here, we revisit the structure/function relationships of this long pentraxin in light of the most recent advances in its structural biology, with a focus on the interplay with complement and the emerging roles as a component of the ECM. Differences to and similarities with the short pentraxins are highlighted and discussed.

## 1 Introduction

The innate immune system is traditionally regarded as the first line of defense against invading pathogens (1). Cellular and molecular effector mechanisms of innate immunity are typically induced upon recognition of PAMPs (pathogen-associated molecular patterns, i.e. motifs shared by evolutionarily close microbial families that are often localized on the cell surface and are essential for fitness) by PRMs (cell-borne or soluble pattern recognition molecules that are expressed both by immune and non-immune cells and act as transducers of activation and modulation signals) (2). Fluid-phase PRMs are regarded as evolutionary ancestors of antibodies in that they exert immunoprotective and immunomodulatory effects by means of opsonic and neutralizing properties, promotion of phagocytosis and complement activation. In addition to their roles in pathogen recognition and disposal, PRMs are increasingly acknowledged as key players in tissue remodeling, whereby they recognize DAMPs (damage/danger-associated molecular patterns) and convey biochemical messages for removal of cellular debris and tissue regeneration (3).

**Figure 1:**
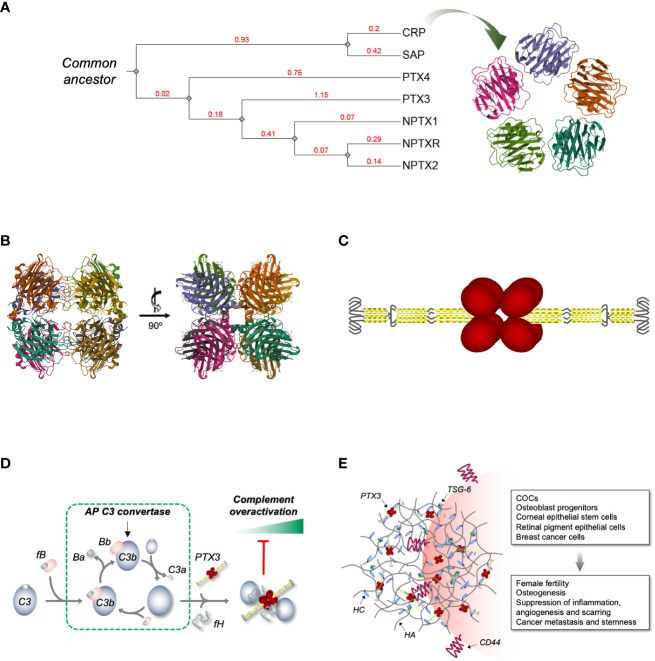
*Phylogenetic relationships across human pentraxins, 3D model of PTX3 and its interplay with complement and HA ECM.* **(A)** Phylogenetic tree of the human PTX family of proteins. The tree was constructed using the Phylogeny.fr web server [http://www.phylogeny.fr/ and (6)], edited and annotated with the iTOL (interactive Tree Of Life) online tool [https://itol.embl.de/ and (7)]. The extent of genetic variation across members of the family (i.e., the average number of amino acid substitutions per site) is indicated by the branch lengths. These are drawn to scale and expressed as arbitrary unit (in red) with tree nodes represented by grey diamonds. The accession numbers used for the analysis are NP_000558.2 (CRP), NP_001630.1 (SAP), NP_002843.2 (PTX3), NP_001013680.1 (PTX4), NP_002513.2 (NPTX1), NP_002514.1 (NPTX2) and NP_055108.2 (NPTXR). Also, a 3D model of human CRP is shown [PDB-ID: 1GNH and (8)] that highlights the symmetric pentameric quaternary structure of this short pentraxin. **(B)** Orthogonal views of a high-resolution model of the C-terminal pentraxin domains of the human PTX3 based on Cryo-EM that show these domains to fold into octamers with D4 symmetry [PDB-ID: 7ZL1 and (9)]. **(C)** Schematic drawing of the PTX3 protein with the N-terminal regions (in yellow) forming two parallel tetrameric coiled coils at the opposite sides of the C-terminal core (in red) [based on (9)]. Hinge and intrinsically disordered regions (represented by black lines) bring flexibility to the structure. **(D)** We have reported that surface bound PTX3 forms a ternary complex with fH and C3b that acts as a “hot spot” for AP inhibition (10). Indeed, when bound to PTX3 and fH, C3b is no longer accessible to factor B (fB, which during AP activation is proteolytically processed to Ba and Bb, the latter being a component of the AP C3 convertase) and loses the ability to amplify the complement cascade (with further cleavage of C3 to C3a and C3b) and the associated inflammatory response (including production of the anaphylatoxin C3a). **(E)** PTX3 is an essential component of the HA-rich ECMs that transiently form in inflammatory and inflammatory-like conditions. Incorporation of PTX3 in these matrices requires synthesis of covalent adducts between HA and heavy chains (HCs) of the proteoglycan IαI (HC•HA), a reaction that is catalyzed by the hyaladherin TSG-6. PTX3 makes multiple, non-covalent interactions with the HC components of the HC•HA complex, and in this way cross-links HA. This mechanism has major implications in female fertility (11) and has been associated with the anti-inflammatory, -angiogenic and -scarring properties of the PTX3/HC•HA complex isolated from the human amniotic membrane (12). Also, through the HA receptor CD44, HA-embedded PTX3 has been recently reported to promote osteogenesis (13) and breast cancer growth, stemness and metastasis (14).

Pentraxins are an evolutionarily conserved family of soluble PRMs that share a common sequence motif (i.e., the pentraxin signature His-x-Cys-x-Ser/Thr-Trp-x-Ser) and typical quaternary structures (4). This comprises short and long pentraxins, each with distinctive structural and functional characteristics. First identified in the ‘30s as an opsonin that recognizes the C-type polysaccharide of *S. pneumoniae* (5), C-reactive protein (CRP) is the prototypical short pentraxin with five identical protomer subunits assembled into symmetric disc-like pentamers stabilized by non-covalent interactions [**Figure 1A** and (15)]. A similar structural organization is found in serum amyloid P component (SAP) (16), another short pentraxin identified in the ‘70s that shares with CRP calcium-dependent recognition of several ligands (17). High levels of sequence and structural homology across short pentraxins from evolutionary distant species suggest that this PRMs play essential roles in innate immunity, even though their functions as opsonizing and complement-fixing molecules have probably become redundant, being overshadowed by other players of the immune system like immunoglobulins (18). Experimental evidence from gene-modified animals indicates that CRP exerts host protective functions in bacterial, especially pneumococcal, infections (19). Importantly, this protein is extensively used in the clinical practice as a non-specific systemic marker of inflammation (20). Serendipitously discovered in studies designed to evaluate the effects of CRP on lymphocytes (18), SAP has been recently acknowledged as an important player in the innate immune reaction to the opportunistic fungal pathogen *A. fumigatus* (21). Also, SAP is long-known as a an essential component of amyloid deposits (22). This property has been clinically exploited to develop SAP-based scintigraphy tracers for *in vivo* imaging of amyloid deposits (23, 24), and combination therapies to target these pathological fibrils in amyloidosis and Alzheimer’s disease (25, 26).

Cloned in the early ‘90s, PTX3 is considered the paradigm of long pentraxins (27). Additional members of this subfamily are pentraxin 4 [PTX4; (28)], neuronal pentraxins 1 (NPTX1 or NP1 (29);) and 2 [NPTX2, also called Narp or NP2; (30)], and the transmembrane protein neuronal pentraxin receptor [NPTXR (31)]; (see **Figure 1A** for an overview of the phylogenetic relationships across human pentraxins). Like all other long pentraxins, the PTX3 protomer contains a compact C-terminal domain with sequence homology to the short pentraxins and an elongated N-terminal region (32–34). Initial functional evidence on PTX3 dates to the early 2000s, when a non-redundant immunoprotective role against *A. fumigatus* was documented by Garlanda et al. (35). Since this original discovery, other functions have emerged for this long pentraxin, including a crucial role in female fertility (11) and extrinsic oncosuppressive effects (36). Here, we reconsider the functional landscape of PTX3 in relation to the protein’s structure, a high-resolution model of which have been recently published. Within this frame, we will discuss emerging vistas on the interplay between PTX3, complement and ECM.

## 2 Structural biology of PTX3

PTX3 is made at sites of infection and inflammation by several immune and non-immune cell types upon stimulation with inflammatory cytokines (i.e., interleukin-1β, IL-1β, and tumor necrosis factor-α, TNF-α), microbial moieties (i.e., lipopolysaccharide, LPS) and intact microorganisms (i.e., *A. fumigatus*) (27). This marks a profound difference to CRP, whose synthesis is mostly induced in the liver by interleukin 6 (IL-6) and acts as a systemic (rather than local) marker of inflammation (37). Cellular sources and gene regulation of PTX3 are extensively reviewed in (27, 38). In this chapter, we will focus on the protein’s structure that, as anticipated by our own work, and refined in recent biophysical studies, marks an additional deviation from short pentraxins.

In 2022 Noone et al. reported the first high-resolution 3D model of PTX3 (first of this kind for a long pentraxin, actually) based on a hybrid cryoelectron microscopy (Cryo-EM)/AlphaFold strategy (9). In this study a Cryo-EM 2.5-Å map of the C-terminal pentraxin domains was generated where these regions folded into a rather unique (compared to the short pentraxins) D4 symmetrical octamer. More precisely, the C-domains were found to form a dimer of tetramers, with each tetramer arranged into a planar ring stabilized by noncovalent interactions, and the two tetramers held together by disulfide bonds (**Figure 1B**). Despite a low level of sequence identity between the C-domain of PTX3 and CRP or SAP (∼28%), these proteins all share a high degree of structural similarity (9). Nonetheless, the short pentraxins fold into pentamers (rather than tetramers; see **Figure 1A**) owing to poor conservation of the inter-subunit interfaces. Also, the metal-binding site present in CRP, SAP (17) and the other long pentraxin NPTX1 (9) is replaced by a disulfide bridge (C317/C318) (33) and an N-glycosylation site (39), which perhaps explains why PTX3 recognizes most of its ligand in a Ca^2+^-independent fashion (27). In the Cryo-EM map two water molecules were found proximal to the His residue of the pentraxin motif, raising the possibility that this residue might establish H-bonds with bulk water and thus act as a sensor of the microenvironment’s pH (9) (an intriguing mechanism, given that acidic pH values have a fundamental impact on the function of PTX3 in the ECM (40); see below).

The cryo-EM map revealed α-helical motifs protruding out of the pentraxin core that were partially resolved. AlphaFold was then used to generate *in silico* predictions of the remaining N-terminal regions and a 3D model was generated that showed the N-domains to form two long tetrameric coiled coils at opposite sides of the C-terminal complex (**Figure 1C**). Each tetramer contains two hinge regions (encompassing inter-chain disulfide bonds), which brings to the protein a high degree of flexibility (9). The two N-domains have a symmetric arrangement [as opposed to the asymmetric model we proposed based on mid-/low-resolution data (34)] and terminate with an intrinsically disordered region (initial 28-30 amino acids), which collectively provides an extended range of motion and possibly the ability to adapt to diverse interaction interfaces.

## 3 Structure/function relationships

The structural complexity of the PTX3 protein perhaps explains the rather vast spectrum of interactions and functions of this pentraxin, which ranges from infection immunity to regulation of inflammation, tissue remodeling and cancer (summarized in **Table 1**).

**Table 1:** Major interactions of PTX3 and their functional outcomes.

	Ligand	Binding interface	Function	*Refs.*
Microorganisms	SARS-CoV-2	Nucleocapsid protein recognition by the PTX3 N-term	Prognostic indicator of short-term mortality in COVID-19	(41–43)
	Influenza virus	Viral hemagglutinin binding to sialic acid residues of the PTX3 C-term	Inhibition of hemagglutination, neutralization of virus infectivity, inhibition of viral neuraminidase	(44)
	Cytomegalovirus	Unknown	Inhibition of viral entry and infectivity *in vitro*, protection from murine CMV infection/reactivation and *A. fumigatus* superinfection *in vivo*	(45)
	*A. fumigatus*	Unknown cell wall ligand binding to the PTX3 N-term	Opsonization, promotion of phagocytosis and killing by neutrophils via complement and Fcγ receptors	(35, 46)
	*K. pneumoniae*	Ca^2+^-dependent recognition of KpOmpA (binding site on PTX3 unknown)	Amplification of the inflammatory response *in vivo*	(47)
	*N. meningitidis*	Ca^2+^-independent binding of meningococcal antigens GNA0667, GNA1030 and GNA2091 to the PTX3 N-term	Amplification of the antibody response and protection from infection *in vivo*	(48)
	*E. coli*	Unknown	Protection from urinary tract infection	(49)
Receptors	P-selectin	P-selectin binding to sialic acid residues of the PTX3 C-term	Inhibition of leukocyte extravasation	(50)
	FcγRIIa and III	Unknown	Promotion of phagocytosis	(46)
	CD44	209-217aa and 352-360aa regions of the PTX3 C-term	Osteogenesis and cancer growth/metastasis and	(13, 14)
Hemostasis	Fibrinogen/Fibrin	pH-dependent binding to the PTX3 N-term	Enhancement of plasmin-mediated fibrinolysis	(40)
	Plasminogen	pH-dependent binding of the KR3-KR5 domains to the PTX3 N-term		
Angiogenesis	FGF2	97-110aa sequence of the PTX3 N-term	Inhibition of angiogenesis, restenosis and cancer progression	(51–55)
	FGF8	Unknown		
Complement	C1q	Sialic acid-dependent binding to the globular head of C1q (mainly through B chain Arg residues)	Control of the CP	(56, 57)
	C3b	Unknown	Inhibition of the AP	(10)
	fH proteins	Ca^2+^- and glycosylation-dependent recognition of CCP7 of fH and FHL-1 (by the PTX3 C-term) and CCP19-20 of fH and FHR1 (by the PTX3 N-term)	Inhibition of the AP	(10, 58–61)
	C4BP	Ca^2+^-dependent recognition of SCRs 1-3 of the C4BP α-chain (binding site on PTX3 unknown)	Inhibition of the CP/LP	(62)
	MBL	Unknown	Cross-activation of the LP	(63)
	Ficolins	Glycosylation-dependent interaction		(64)
ECM	IαI	Mg^2+^-dependent binding of the PTX3 N-term to HCs 1 and 2 of IαI	Assembly and stability of the HA ECM	(33, 65–67)
	TSG6	Link module recognition by the PTX3 N-term	Assembly and stability of the HA ECM and regulation of angiogenesis	(55, 67)
	TSP1	C-terminal globular domain (E123CaG-1) binding to the PTX3 N-term	Control of synaptogenesis	(68)
Others	Histones	N-term domain of histones (binding site on PTX3 unknown)	Protective functions against extracellular histone-mediated cytotoxicity	(32)

PTX3 acts as host protective soluble PRM towards a number of fungal, bacterial, and viral pathogens, including *A. fumigatus* (35, 46), *S. pneumoniae* (69), uropathogenic *E. coli* (49), influenza virus (44) and cytomegalovirus (45). Locally induced at sites of infection, PTX3 opsonizes cognate microorganisms upon engagement of selected PAMPs [e.g., outer membrane protein A, OmpA, of *K. pneumoniae* (47), outer membrane vesicles, OMV, and meningococcal antigens of *N. meningitidis* (48), nucleocapsid protein of SARS_COV_2 (41)] and promotes viral neutralization as well as fungal and bacterial phagocytosis and killing (by professional phagocytes, mostly neutrophils). The latter processes imply a tight interaction with complement components (discussed below) and Fcγ-receptors [reviewed in (70)].

Several studies point to PTX3 as a modulator of inflammation in sterile (in addition to infectious) settings. In this regard, PTX3 is known to interact with P-selectin in a glycosylation-dependent manner and dampen, via a negative feedback mechanism, the P-selectin-mediated extravasation of neutrophils to sites of tissue damage (50). Also, PTX3 participates in the clearance of apoptotic cells and debris (including histones) by modulating their uptake by dendritic cells (71) and acting as an “eat me” tag for late apoptotic neutrophils (72), in addition to regulating complement activation on dying cells (73). Moreover, it tames complement-dependent, tumor-promoting inflammation (36).

PTX3 is acknowledged as an important player in tissue remodeling and cancer. As for this, it is known to sequester FGF2 and FGF8 through its N-terminal region and inhibit their angiogenic and inflammatory activities in models of neovascularization and FGF-dependent tumorigenesis (51–55). Also, an interaction has been suggested for PTX3 with DC-SIGN (dendritic cell-specific intercellular adhesion molecule-3-grabbing nonintegrin) that might be relevant to leukocyte activation and differentiation to fibrocyte (74), which however requires experimental validation. Other than FGFs, other ligands have been proposed for PTX3 that are relevant to cancer biology, including components of the complement system and CD44 (see below) with however conflicting outcomes (see (75) for further discussion). Finally, PTX3 is known to intercept major mechanisms of fibrin- and HA-remodeling which will be discussed in a separate paragraph.

### 3.1 Crosstalk with the complement system

As “ante-antibodies”, pentraxins functionally cooperate with complement, *de facto* extending and modulating both recognition and effector phases of this system (76). The first complement ligand of CRP and SAP to be identified was C1q, whose interaction with these short pentraxins results into activation of the classical pathway (CP) (77, 78). Follow-up studies revealed that components of the lectin pathway (LP) too, with major regard to ficolins, form complexes with CRP and SAP that favor complement-mediated disposal of apoptotic cells and microbial pathogens (75). Importantly, both CP and LP cooperate with pentraxins through additive and synergistic effects that broaden the repertoire of PAMPs/DAMPs recognition and effector functions of the humoral innate immunity [reviewed in (79)]. Despite fundamental immunoprotective functions, over-activation of the complement system can be pathogenic. In this regard, short pentraxins have been proposed to play a dual role. For example, in myocardial infarction and ischemic stroke CRP binds DAMPs on injured cells and exacerbates complement-dependent tissue damage (80). On the other hand, short pentraxins have been involved in suppressive pathways that limit this process by recruiting complement inhibitors, like C4b-binding protein [C4BP, major soluble inhibitor of the CP and LP that is recognized by SAP (81)] and factor H [fH, primary fluid phase inhibitor of the alternative pathway, AP, that is bound by CRP (82)].

Like the short pentraxins, PTX3 tightly cross talks to the three pathways of complement with varying and context-dependent outcomes (83). For example, recognition units of the LP (i.e., mannose-binding-lectin, MBL, and ficolins) form with PTX3 hetero-complexes with LP amplifying activity towards fungal pathogens, like *C. albicans* (63) and *A. fumigatus* (64). However, the interaction of PTX3 with C1q (56) results into either activation (on surfaces) or inhibition (in solution) of the CP (57). Also, this long pentraxin is known to interact with complement inhibitors and restrain overactivation of this system. In this regard, PTX3 recruits C4BP to sites of tissue remodeling, including ECM and apoptotic cells, and inhibit activation of the CP/LP (62). More importantly, PTX3 is a ligand of members of the factor H family of proteins (i.e., fH, factor H-like protein 1, FHL-1, and factor H-related proteins 1 and 5, FHR-1 and -5) that collectively control the AP (58–61). This has implications in complement-driven cancer-related inflammation (36), opsonophagocytosis of *A. fumigatus* (46) and *P. aeruginosa* (84), atypical hemolytic uremic syndrome (aHUS) (60), and age-related macular degeneration (AMD) (85).

The retina is emerging as an important stage for the complement-modulating properties of PTX3. Indeed, this protein has been localized within and around the ECM of the outer blood-retinal barrier (oBRB), including the Bruch’s membrane (BrM), retinal pigment epithelium (RPE), and choriocapillaris, major sites of complement dysregulation in AMD (86). We recently reported that PTX3 forms a ternary complex with C3b (component of the AP C3 convertase) and fH on acellular surfaces (that mimic the BrM) and acts as a “molecular trap” for AP activation (10) (**Figure 1D**). Also, we documented that PTX3 interacts with FHL-1 (a truncated form of fH that comprises complement control proteins, CCPs, 1-7 and retains the ability to inhibit the AP), and this interaction is affected by the Y402H polymorphism (a major AMD-associated allele that maps in CCP7 and is thus present both in fH and FHL-1) (61). Given that FHL-1 is a primary inhibitor of the AP in the oBRB and displays Y402H-dependent binding to the BrM (87), we postulate that PTX3 exerts BrM (i.e., ECM) anchoring properties towards FHL-1 (in addition to fH) and these might compensate the pathological effects of the 402H allele (88). It is therefore conceived that this long pentraxin participates in the mechanisms of complement homeostasis in the eye whereby its multimeric and flexible structure allows at a time incorporation in the oBRB ECM and retention of complement inhibition. In this regard, it is worth reiterating here that ECM-embedded PTX3 exerts inhibitory (rather than activating) functions towards the CP/LP via a specific interaction with C4BP (62).

### 3.2 Roles in the ECM

In a pivotal study by Doni et al, primary components of hemostasis were identified as high affinity ligands of PTX3, i.e. fibrinogen/fibrin (FG) and plasminogen (PG) (40). These recognize non-overlapping sites in the PTX3 N-terminal domain in a calcium- and glycosylation-independent manner, which allows formation of a tripartite PTX3/FG/PG complex with fibrinolytic activity (in the presence of plasminogen activators). In an analogy with the AP components C3b and fH (10), PTX3 acted as a molecular scaffold to favor the interaction of PG with FG and ensure timely degradation of the fibrin clot (40). Consistent with this view, this long pentraxin had remodeling activity in several models of tissue injury, including skin wound, liver and lung damage, and arterial thrombosis. This activity is distinctive of PTX3 and its N-terminal domain (the short pentraxins lacks recognition of FG and PG) and, more importantly, is pH-dependent, whereby an acidic environment sets the PRM PTX3 in a tissue repair mode (40).

Back in 2002, Varani et al. reported that PTX3 deficiency is associated with severe subfertility in female mice (89). In a follow up study, Salustri et al. demonstrated that this is due to instability of the hyaluronic acid (HA) matrix that forms around the oocyte and the surrounding cumulus cells (a multicellular assembly known as cumulus oophorous complex, COC) prior to ovulation and is necessary for fertilization *in vivo* (11). More importantly, these preclinical findings were corroborated by epidemiological data, whereby *PTX3* polymorphisms have been associated with frequency of offspring (90) and dizygotic twinning (91) in sub-Saharan females. The mechanisms underlying the role of PTX3 in fertility are paradigmatic examples of the structure/function complexity of this protein. In fact, in the preovulatory period the COC-associated HA undergoes physical and chemical remodeling due to the action of inter-α-trypsin inhibitor (IαI), a serum proteoglycan composed of two heavy chains (HCs) that enters the follicle due to permeabilization of the blood/follicle barrier (92), and the HA-binding protein TSG-6 (93), which is locally expressed by follicular cells and catalyzes the covalent transfer of HCs onto HA to form HC•HA complexes (94). These HC•HA adducts act as scaffolds for incorporation of the PTX3 protein that is secreted by cumulus cells upon stimulation with oocytic factors and second messengers (89). Owing to its multimeric structure, PTX3 has multiple Mg^2+^-dependent binding sites (in the N-domain) for the HC components of HC•HA, and thus acts as a node in crosslinking HA, providing stability to the HA ECM (33, 65–67) (**Figure 1E**). Beside ovulation, these mechanisms might be relevant to pathology, including inflammatory and infectious diseases of the bone (95), joint (96) and lung (97), and are distinctive of PTX3, again due to lack of the N-terminal domain in CRP and SAP. Also, HA-embedded PTX3 has been recently proposed as a promoter of synaptogenesis in the developing central nervous system, where it forms a rheostat with astrocyte-derived thrombospondin 1 (TSP1) (68).

Not only incorporation of PTX3 into HA-rich ECMs has scaffolding implications, but it also appears to convey intracellular signals. In this regard, PTX3 is present in the HA-dependent pericellular matrix of MC3T3-E1 osteoblasts, where it promotes a self-sustained osteogenic program in inflammatory conditions through a functional axis comprising HA, CD44 (major cellular receptor for HA) and the activated focal adhesion kinase (FAK)/protein kinase B (AKT) signaling cascade (13). On the same line, HA/HCs/PTX3 complexes isolated from the human amniotic membrane suppresses inflammation, angiogenesis and scarring in preclinical models of corneal and retinal pathology (12). Also, in a recent report by Hsiao et al, a direct interaction has been documented between CD44 and PTX3 that activates ERK1/2, AKT and NF‐κB pathways and contributes to metastasis/invasion and stemness of a triple‐negative breast cancer cell line (14) (see **Figure 1D**). It is not clear whether embedding into the HA pericellular matrix is required for PTX3 to recognize CD44, however, given that the CD44-binding interface is in the C-terminal domain (14), it is conceivable that, when incorporated into HA-ECMs [through its N-terminal region (66)], PTX3 retains binding to CD44 while modulating (e.g., via HA-crosslinking) its interaction with HA. This might have implications in leukocyte adhesion and activation (98), in addition to cancer metastasis and stemness (99).

## 4 Conclusion and perspectives

PTX3 has been implicated in various pathological conditions, including infections (100), cardiovascular diseases (101), bone disorders (95) and cancer (36, 102), where it has potential as a diagnostic and/or prognostic marker and therapeutic target. Importantly, the plasmatic levels of this pentraxin have been consistently associated with disease’s severity and outcome in sepsis and septic shock, tuberculosis, dengue and meningitis [reviewed in (100)], and, more recently, COVID-19 (42, 43). Also, polymorphisms in the *PTX3* gene have been associated with the protein’s levels in the plasma and the risk of developing selected opportunistic fungal and bacterial infections [reviewed in (27)]. In this respect, the rs2305619, rs3816527 and rs1840680 single nucleotide polymorphisms (SNPs) form a common haplotypic block where the second SNP causes an amino acid substitution (Asp to Ala) at position 48 in the N-terminal domain. This polymorphism does not alter the protein structure substantially (56), neither does it affect the interaction of PTX3 with C1q (56) and *A. fumigatus* (103), however it is at present unknown whether it has any impact on the recognition of other ligands, with major regard to the complement proteins fH and C3b, and the incorporation of PTX3 in HA- and/or fibrin-rich ECMs.

CRP is widely used as a clinical biomarker to assess inflammation and predict the risk of cardiovascular diseases (104). Comparative analysis of the structure/function relationships of short and long pentraxins offers unprecedented opportunities to understand the roles of these PRMs in the immune response and their implications in the pathogenesis of several diseases. Recent insights into the structure of PTX3 (the first long pentraxin to be unraveled in such detail) have shed light on its unique architecture and pinpointed the molecular determinants of its immune-modulatory functions (9). In this regard, multi-domain composition of the protomer subunits, glycosylation and quaternary organization collectively contribute to the diverse ligand recognition and interaction capabilities of this long pentraxin. Here we highlighted emerging aspects of the PTX3 biology that intercept fundamental processes of inflammation and tissue remodeling and have translational value in clinical settings that are characterized by dysregulation of complement activation and ECM turnover.

## Author contributions

AM: Data curation, Writing – original draft. FC: Data curation, Writing – original draft. BB: Writing – review & editing. AI: Conceptualization, Data curation, Funding acquisition, Project administration, Supervision, Validation, Visualization, Writing – original draft, Writing – review & editing.

## Glossary

**Table:**

aHUS	atypical hemolytic uremic syndrome
AKT	protein kinase B
AMD	age-related macular degeneration
BrM	Bruch’s membrane
C4BP	C4b-binding protein
CCPs	complement control protein
COC	cumulus oophorous complex
CRP	C reactive protein
Cryo-EM	cryogenic electron microscopy
DAMPs	danger-associated molecular patterns
DC-SIGN	dendritic cell-specific intercellular adhesion molecule-3-grabbing nonintegrin
ECM	extracellular matrix
ERK1/2	extracellular signal-regulated kinase 1/2
FAK	focal adhesion kinase
fB	factor B
FG	fibrinogen/fibrin
FGFs	fibroblast growth factors
fH	factor H
FHL-1	factor H-like protein 1
FHR-1 & -5	factor H-related protein 1 & 5
HA	hyaluronic acid
HCs	heavy chains
IL-1β	interleukin-1β
IαI	inter-alfa-trypsin inhibitor
LPS	lipopolysaccharide
MBL	mannose binding lectin
NF‐κB	nuclear factor kappa-B
NPTX1 or NP1	neuronal pentraxins 1
NPTX2 or Narp or NP2	neuronal pentraxins 2
NPTXR	neuronal pentraxin receptor
oBRB	outer blood-retinal barrier
OmpA	outer membrane protein A
OMV	outer membrane vesicles
PAMPs	pathogen-associated molecular patterns
PG	plasminogen
PRMs	pattern recognition molecules
PTX3	pentraxin 3
PTX4	pentraxin 4
RPE	retinal pigment epithelium
SAP	serum amyloid P component
SARS_COV_2	severe acute respiratory syndrome coronavirus 2
TNF-α	tumor necrosis factor-α
TSG-6	tumor necrosis factor-inducible gene 6 protein
TSP1	thrombospondin 1
